# The predictive and prognostic effects of PD-L1 expression on TKI treatment and survival of EGFR-mutant NSCLC: A meta-analysis

**DOI:** 10.1097/MD.0000000000027038

**Published:** 2021-08-27

**Authors:** Bo Lan, Yongfang Wang, Jingni Wu, Kai Wang, Pingli Wang

**Affiliations:** a Department of Respiratory Medicine, The Third People's Hospital of Hangzhou, Hangzhou, China; b Department of Allergy, Second Affiliated Hospital, School of Medicine, Zhejiang University, Hangzhou, China; c Department of Respiratory Medicine, Second Affiliated Hospital, School of Medicine, Zhejiang University, Hangzhou, China; d Department of Respiratory Medicine, Forth Affiliated Hospital, Z School of Medicine, Zhejiang University, Jinhua, China.

**Keywords:** epidermal growth factor receptor, nonsmall cell lung cancer, programmed death-ligand 1, tyrosine kinase inhibitor

## Abstract

Whether programmed death-ligand 1 (PD-L1) expression could predict the outcome of tyrosine kinase inhibitor (TKI) treatment and prognosis of epidermal growth factor receptor (EGFR)-mutant nonsmall cell lung cancer (NSCLC) is remaining controversial.

Potential studies were search from PubMed, Embase, and Web of Science databases. Pooled odds ratio of objective response rate was used to describe the relationship between PD-L1 expression and primary resistance to EGFR-TKIs. Pooled hazard ratios (HRs) of progression-free survival (PFS) and overall survival (OS) were included to assess the effects of PD-L1 status on the outcome of EGFR-TKI treatment and survival of EGFR-mutant NSCLCs.

Eighteen eligible studies (1986 EGFR-mutant NSCLCs) were included in this meta-analysis. Positive PD-L1 expression correlated with lower objective response rate of EGFR-TKI treatment (odds ratio [95% confidence interval {CI}] = 0.52 [0.28–0.98], *P* = .043), while PFS (adjusted HR [95% CI] = 1.49 [0.96–1.89], *P* = .332) and OS (HR [95% CI] = 1.24 [0.70–2.20], *P* = .456) of EGFR-TKI treatment did not correlated with PD-L1 status. Furthermore, PD-L1 expression was not a predictive biomarker for the OS (HR [95% CI] = 1.43 [0.98–2.08], *P* = .062) in overall EGFR-mutant cohort.

Positive PD-L1 expression indicated a higher incidence of primary resistance, but did not correlate with the PFS or OS of EGFR-TKI therapy. In addition, PD-L1 expression was unlikely a predictive biomarker for prognosis of EGFR-mutant NSCLCs.

## Abstract 2

Supplemental Digital Content is available in the text

## 1 Introduction

Although there has been tremendous progress in cancer therapy in recent decade, lung cancer is remaining the leading cause of cancer-related death worldwide.^[1]^ Management of patients with advanced-stage nonsmall cell lung cancer (NSCLC) has been revolutionized as a result of development in molecular-targeted therapy and immunotherapy.^[2,3]^ Approximately 50% of Asians and 10% to 15% Caucasians harbor activating mutation of epidermal growth factor receptor (EGFR), the first oncogenic driver discovered.^[4,5]^ Several randomized controlled trials have demonstrated a significant superior efficacy of first-line tyrosine kinase inhibitor (TKI) treatment to conventional platinum-based chemotherapy in EGFR-mutant cohort.^[6,7]^ However, nearly 30% of NSCLCs harboring EGFR activation show primary resistance to EGFR-TKIs and nearly all of the EGFR-TKI responders develop an acquired resistance within 1 year of treatment.^[8,9]^ The multiple mechanisms of primary/acquired resistance include deletion polymorphism of Bcl-2 family member (BIM), alteration of TGF-β/Smad signaling pathway, activation of parallel or downstream signaling pathway, second site mutations (T790M mutation) and histological transformation.^[10,11]^ Nevertheless, half of primary resistance and 25% to 30% of acquired resistance mechanisms are not fully understood yet.^[9]^ Therefore, further study for the resistance mechanisms of EGFR-TKIs is urgently needed.

In addition to molecular-targeted therapy, immunotherapy targeting the programmed death-1 (PD-1)/programmed death-ligand 1 (PD-L1) axis is another tremendous progress in management of advanced-stage NSCLCs.^[12–15]^ Tumor PD-L1 expression assessed by immunohistochemical (IHC) method shows a significant correlation with the response of anti-PD-1/PD-L1 therapy, and higher level of PD-L1 is confirmed to predict a better outcome.^[16]^ Interestingly, increasing evidences indicate an internal correlation between PD-1/PD-L1 axis and activation of EGFR oncogene in NSCLC.^[17,18]^ Preclinical studies demonstrate PD-L1 expression could be significantly upregulated in bronchial epithelial cells with mutant-EGFR expression, while EGFR-TKI treatment results in a downregulation of PD-L1 in NSCLC cell lines harboring EGFR activation.^[19]^ However, in clinical practice, uninflamed phenotype and weak immunogenicity of EGFR-mutant NSCLCs lead to poor outcome with significant lower objective response rates (ORRs) and shorter progression free survival (PFS) to anti-PD-1/PD-L1 therapy.^[20]^ Furthermore, upregulation of PD-L1 is observed in the context of acquire EGFR-TKI resistance, indicating that immune mechanism may contribute to the resistance of molecular-targeted therapy.^[21,22]^

However, previous studies exploring the predictive and prognostic values of PD-L1 expression in EGFR-mutant NSCLCs treated with TKIs have yielded paradox conclusions as a consequence of different prior treatment status, cutoff value for defining PD-L1 positive, IHC assay and etc. For example, study from Lin et al^[23]^ indicates positive PD-L1 expression might be a favorable biomarker candidate for the outcome of EGFR-TKI treatment. While Yang et al^[24]^ demonstrate higher PD-L1 expression indicating a poorer response to EGFR-TKI in NSCLC. Basing on the present evidences, we hypothesize PD-L1 might potentially predict the patients’ sensitivity to EGFR-TKIs and affect the long-term outcome of patients receiving EGFR-TKI therapy.

Before our study, 2 meta-analysis studies on this issue have been published in the past.^[25,26]^ However, these 2 studies provide weak evidences because of fewer included studies and lack of subgroup analysis detecting the sources of large heterogeneity. Thus, we conduct this study to determine whether tumor PD-L1 expression associates with efficacy of EGFR-TKI treatment. Demonstrating the relationship between PD-L1 status and resistance to EGFR-TKIs might be helpful to predict the resistance among NSCLCs treated with EGFR-TKIs in advanced. The primary objective of this meta-analysis is to evaluate the predictive values of PD-L1 expression in EGFR-TKI responsiveness including ORR, PFS, and overall survival (OS). The secondary objective is to explore the relationship between PD-L1 expression and prognosis of EGFR-mutant NSCLCs.

## 2 Method

### 2.1 Search strategy

The potential studies in PubMed, Embase, and Web of Science databases, published up to June 15, 2020, were reviewed in this meta-analysis. We searched potential studies by using a combination of “lung cancer” and “EGFR” and “PD-L1” with their related words (Tables S1–S3, Supplemental Digital Content, http://links.lww.com/MD2/A346, http://links.lww.com/MD2/A347, http://links.lww.com/MD2/A348, literature search strategy used in Pubmed, Embase, and Web of Science). This meta-analysis was performed in accordance with the meta-analysis of observational studies in epidemiology compliant. The study does not require ethical approval as the meta-analysis is based on published research, and the original data are anonymous.

### 2.2 Selection and exclusion criteria

Two independent reviewers were responsible for study assessment, while any disagreements between the first 2 reviewers were resolved by a third investigator. Literature selecting criteria were listed as follows: included cases with pathological diagnosis of EGFR-mutant NSCLC; PD-L1 expression was assessed under IHC method and categorized as positive or negative; studies reported survival outcome for PD-L1 positive versus PD-L1 negative group as hazard ratios (HRs) with 95% confidence intervals (CIs) or in Kaplan–Meier (KM) curves; and studies reported the prevalence of PD-L1 expression in primary resistance cases. Studies were excluded while the following exclusion criteria were met: case reports, duplicate publications, reviews, editorials, and expert opinions; PD-L1 expression was not accessed under IHC method; EGFR-TKIs were treated as adjuvant therapy; and studies was not published in English language.

### 2.3 Extraction of data and assessment of quality

Essential information of each eligible study was extracted by 2 independent investigators: name of first author; year of publication; geographic region; assays used for IHC assessment; number of EGFR-mutant cases; cutoff value for defining PD-L1 positive expression; types of TKI; line of TKI treatment; and outcome assessment measured by ORR, PFS, and OS. Both PFS and OS were included as the HRs with 95% CIs obtained by comparing the 2 groups (PD-L1 positive versus PD-L1 negative). When HR for PFS or OS was not available, the KM curves were digitalized by Engauge Digitizer 4.1 software, and the HR was estimated from the recalculated KM curves using the approach described by Guyot et al.^[27]^ Each estimated HR was calculated twice independently to ensure consistency of the results. Furthermore, comparison of ORR between PD-L 1 positive and negative group was described by odds ratio (OR) with 95% CI.

The Newcastle–Ottawa Scale with a maximal score of 9 was applied for quality assessment of each eligible studies.^[28]^ Studies with a final score of 8 to 9 were identified as “high quality”, while studies with a score lower than 5 were identified as “low quality” and the rest were identified as “moderate quality”.

### 2.4 Statistical analysis

Data synthesis and analysis was conducted with the software STATA 14.0. Heterogeneity among eligible studies was measured by Q test and *I*^*2*^ statistic, while the *P* value of Q test was ≤.10 and/or *I*^*2*^ statistic was >50% indicated a significant level of heterogeneity. A random-effects model would be adopted to estimate the pooled HR or OR with 95% CI when significant heterogeneity was identified. Otherwise, the fixed-effects model would be adopted. In this meta-analysis, *P* < .05 represented a statistical significance. Both Begg test and Egger test were adopted to detect publication bias when more than 10 studies included, and *P* < .10 indicated a significant publication bias.^[29]^ When significant publication bias was presented, we conducted a trim-and-fill method to estimate the amount of hypothetical missing studies and adjust the pooled results. The sensitivity analysis by sequentially deleting 1 study at a time was adopted to explore the stability of the pooled results and the origin of heterogeneity. In addition, subgroup analysis stratified by IHC assays, cutoff standards for PD-L1 positive and line of TKI treatment was performed to further explore the sources of heterogeneity and the variation of the predictive and prognostic values of PD-L1 among different situations.

## 3 Results

### 3.1 Search results and characteristics of studies

**Figure 1:**
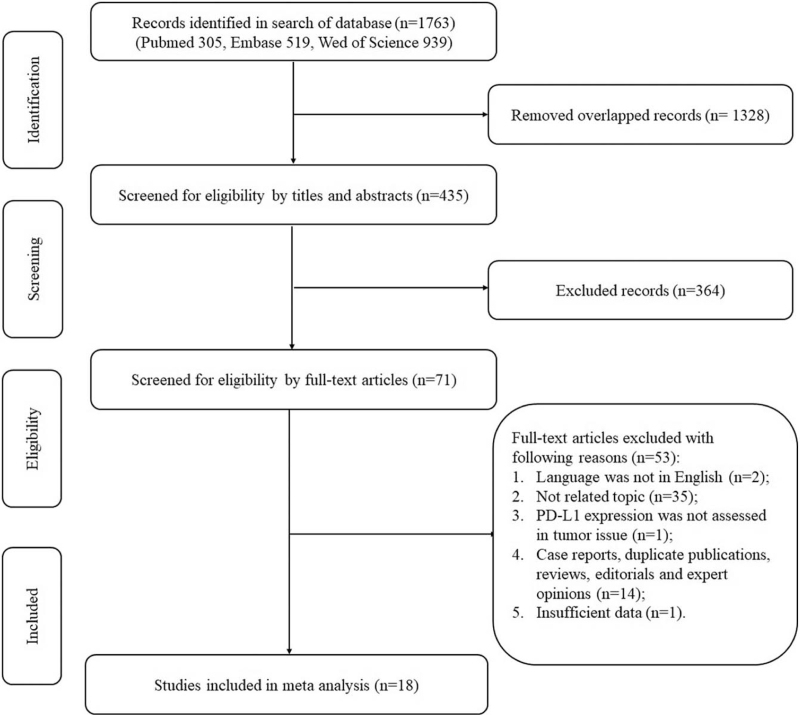
Flow diagram of the literature selecting procedure. PD-L1 = programmed death-ligand 1.

The search of 3 databases identified 1763 records. After records screening and full-text assessments, 18 studies with 1986 EGFR-mutant NSCLCs were finally included in this meta-analysis. Literature selection procedure was presented by flow diagram in detail (Fig. 1). Among the included studies, 17 of them were conducted on East-Asian population, while only 1 study was conducted on Caucasian population. Twelve studies (14 cohorts) explored the relationship between PD-L1 and EGFR-TKI responsiveness including ORR, PFS, and OS in NSCLCs of advanced-stages (stage IIIB–IV). For cutoff standards, 1% tumor cells with positive staining was most commonly used. About quality assessment, 4 and 14 studies were classified as “high-quality” and “moderate-quality” respectively. Main characteristics of each eligible study was summarized (Table 1).

**Table 1:** Main characteristics of included studies in the meta-analysis.

Author	Year	Region	EGFRm + cases	TKIs	Line of TKI treatment	PD-L1 assay	Cutoff	Outcome	NOS score
D’lncecco^[41]^	2015	Italy	55	G/E	1st/≥2nd	ab58810	5%	PFS, OS	6
Tang^[42]^	2015	China	99	G/E	1st/≥2nd	E1L3N	5%	ORR, PFS, OS	7
Lin^[23]^	2015	China	56	G/E	1st/≥2nd	ab58810	mean H score	ORR, PFS, OS	7
Mori^[43]^	2016	Japan	136	NR	NR	EPR1611	50 PD-L1 score	OS	6
Cho^[44]^	2017	Korea	319	NR	NR	22C3	1%, 50%	OS	8
Kim^[45]^	2017	Korea	69	NR	1st/≥2nd	22C3	1%	PFS	8
Soo^[46]^	2017	Singapore	70	NR	1st	SP142	mean H score	PFS, OS	5
Liu^[47]^	2018	China	376	NR	NR	SP142	25%	OS	5
Su^[48]^	2018	China	84	NR	1st	SP142	5%, 50%	ORR, PFS	8
Kobayashi^[49]^	2018	Japan	32	first generation	1st	NR	5%	ORR, PFS, OS	6
Bai^[34]^	2018	China	73	NR	NR	E1L3N	5%	OS	8
Takashima^[50]^	2018	Japan	84	NR	1st	SP142	1%	ORR	5
Yoneshima^[51]^	2018	Japan	80	NR	1st	22C3	1%	PFS	7
Hsu^[52]^	2018	China	57	G/E/A	1st	SP263	1%	ORR, PFS, OS	6
Siripoon^[53]^	2018	Thailand	125	NR	NR	22C3	1%	OS	5
Matsumoto^[54]^	2019	Japan	52	G/E/A	1st	28-8	50%	ORR, PFS	7
Yang^[24]^	2020	China	153	G/E/A	1st/≥2nd	22C3	1%, 50%	ORR, PFS, OS	7
Kim^[55]^	2019	Korea	66	G/E/A	1st/≥2nd	SP142/SP263/22C3	1%	PFS, OS	6

A = represents afatinib, E = represents erlotinib, EGFR = epidermal growth factor receptor, G = gefitinib, NOS = Newcastle–Ottawa Scale, NR = represents “not reported”, ORR = objective response rate, OS = overall survival, PD-L1 = programmed death-ligand 1, PFS = progression-free survival, TKI = tyrosine kinase inhibitor.

### 3.2 Positive PD-L1 expression indicated a higher incidence of primary resistance to EGFR-TKIs

**Figure 2:**
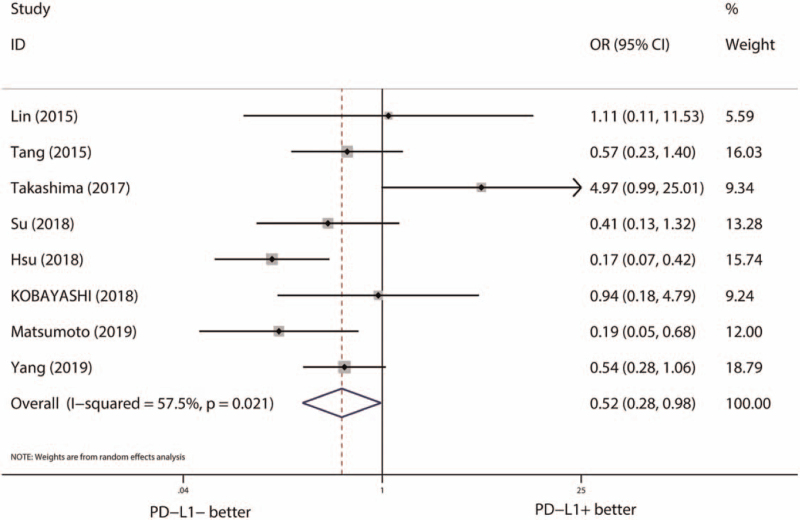
Pooled effects of PD-L1 expression on ORR of EGFR-TKI treatment. CI = confidence interval, EGFR = epidermal growth factor receptor, OR = odds ratio, ORR = objective response rate, PD-L1 = programmed death-ligand 1, TKI = tyrosine kinase inhibitor.

To explore the relationship between PD-L1 expression and primary resistance to EGFR-TKIs, we compared the ORR of EGFR-TKI treatment among PD-L1 positive and PD-L1 negative group. Eight studies with 700 EGFR-mutant NSCLCs were included, and significant heterogeneity was identified among included studies (*I*^*2*^ = 57.5%, *P* = .021). Adopting a random effect model, pooled OR indicated the ORR of EGFR-TKI treatment was statistically significant lower in PD-L1 positive group (OR [95% CI] = 0.52 [0.28–0.98], *P* = .043) (Fig. 2). In addition, result of sensitivity analysis indicated the study from Takashima et al significantly affected the stability of pooled OR, and positive PD-L1 expression still indicated a lower ORR of EGFR-TKI treatment when we removed this study (Figure S1, Supplemental Digital Content, http://links.lww.com/MD2/A338, sensitivity analysis of the relationship between PD-L1 expression and ORR of EGFR-TKI treatment).

### 3.3 Predictive effects of PD-L1 expression on the clinical outcome of EGFR-TKIs

**Figure 3:**
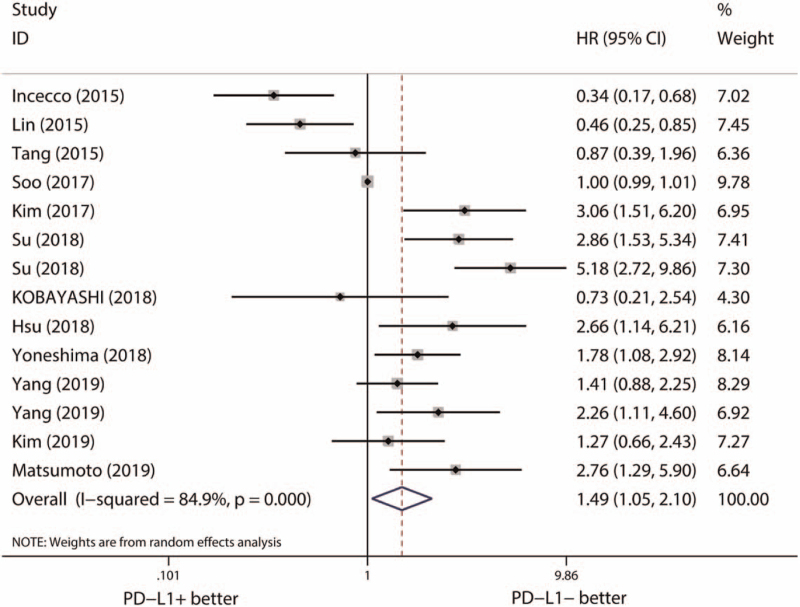
Pooled effects of PD-L1 expression on PFS of EGFR-TKI treatment. CI = confidence interval, EGFR = epidermal growth factor receptor, HR = hazard ratio, PFS = progression-free survival, PD-L1 = programmed death-ligand 1, TKI = tyrosine kinase inhibitor.

**Figure 4:**
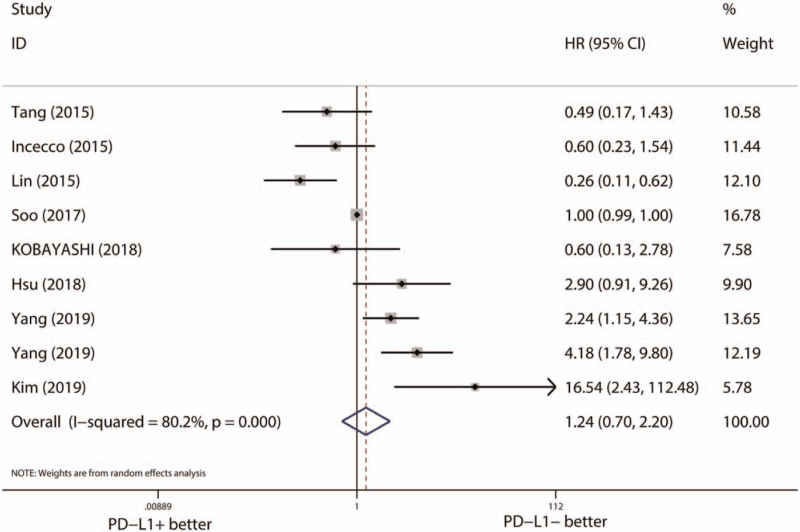
Pooled effects of PD-L1 expression on OS of EGFR-TKI treatment. CI = confidence interval, EGFR = epidermal growth factor receptor, HR = hazard ratio, OS = overall survival, PD-L1 = programmed death-ligand 1, TKI = tyrosine kinase inhibitor.

Then, we evaluated the predictive effects of PD-L1 expression on the clinical outcome of EGFR-TKIs. For PFS, 12 studies (14 cohorts) with 872 EGFR-mutant NSCLCs were included. The included studies showed a significant level of heterogeneity (*I*^*2*^ = 84.9%, *P* < .001). Pooled results with a random effect model demonstrated a significant longer PFS in PD-L1 negative group (HR [95% CI] = 1.49 [1.05–2.10], *P* = .024) (Fig. 3). However, inconsistent results of Begg test (*P* = .913) (Figure S2, Supplemental Digital Content, http://links.lww.com/MD2/A339, publication bias of the relationship between PD-L1 expression and PFS of EGFR-TKI treatment estimated by Begg test) and Egger test (*P* = .071) (Figure S3, Supplemental Digital Content, http://links.lww.com/MD2/A340, publication bias of the relationship between PD-L1 expression and PFS of EGFR-TKI treatment estimated by Egger test) suggested potential publication bias was existed. Subsequently, we quantified the effects of PD-L1expression on the PFS of EGFR-TKI treatment by using the trim-and-fill method, 1 hypothetical missing study was added, and the adjusted result indicated no significant difference of PFS between PD-L1 positive and negative group (HR [95% CI] = 1.49 [0.96–1.89], *P* = .332). Results of sensitivity analysis were consistent with the adjusted result of pooled HR (Figure S4, Supplemental Digital Content, http://links.lww.com/MD2/A341, sensitivity analysis of the relationship between PD-L1 expression and PFS of EGFR-TKI treatment). For OS, 8 studies (9 cohorts) with 588 EGFR-mutant NSCLCs were included. Significant level of heterogeneity (*I*^*2*^ = 80.2%, *P* < .001) was observed. Within a random effect model, PD-L1 expression was not associated with the OS of EGFR-TKI therapy (HR [95% CI] = 1.24 [0.70–2.20], *P* = .456) (Fig. 4). Limited stability found by sensitivity analysis suggested the expression of PD-L1 expression was not a reliable predictor of the OS neither (Figure S5, Supplemental Digital Content, http://links.lww.com/MD2/A342, sensitivity analysis of the relationship between PD-L1 expression and OS of EGFR-TKI treatment).

### 3.4 Prognostic values of PD-L1 expression in overall EGFR-mutant cohort

**Figure 5:**
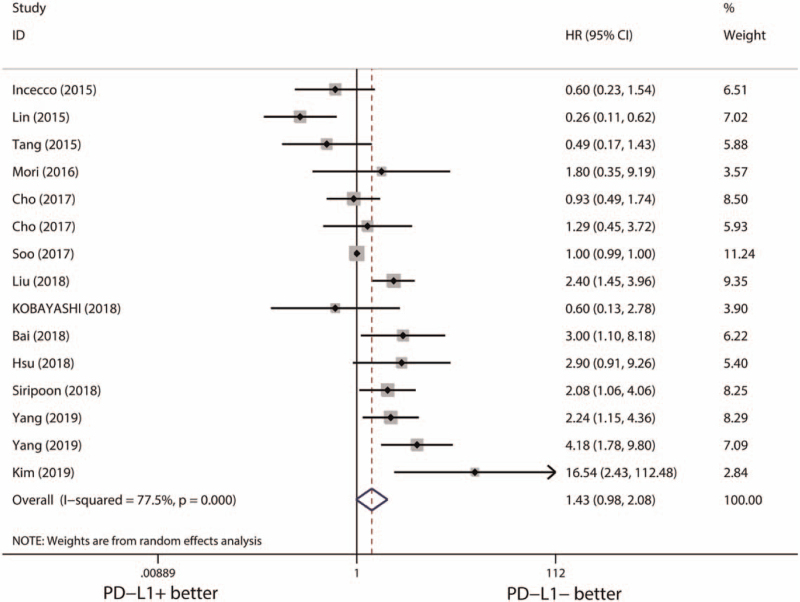
Pooled effects of PD-L1 expression on OS of whole EGFR-mutant cohort. CI = confidence interval, EGFR = epidermal growth factor receptor, HR = hazard ratio, OS = overall survival, PD-L1 = programmed death-ligand 1.

Finally, the prognostic values of PD-L1 expression in EGFR-mutant NSCLCs were explored. Thirteen studies (15 cohorts) with 1617 EGFR-mutant NSCLCs were totally included. Significant heterogeneity was identified (*I*^*2*^ = 77.5%, *P* < .001), and pooled results with a random effect model indicated no association between PD-L1 expression and the OS in EGFR-mutant NSCLC (HR [95% CI] = 1.43 [0.98–2.08], *P* = .062) (Fig. 5). The results of Begg test (*P* = .961) (Figure S6, Supplemental Digital Content, http://links.lww.com/MD2/A343, publication bias of the relationship between PD-L1 expression and OS in EGFR-mutant NSCLCs estimated by Begg test) and Egger test (*P* = .103) (Figure S7, Supplemental Digital Content, http://links.lww.com/MD2/A344, publication bias of the relationship between PD-L1 expression and OS in EGFR-mutant NSCLCs estimated by Egger test) showed an acceptable publication bias. Meanwhile, sensitivity analysis also suggested PD-L1 expression had a limited influence on the OS of EGFR-mutant NSCLCs (Figure S8, Supplemental Digital Content, http://links.lww.com/MD2/A345, sensitivity analysis of the relationship between PD-L1 expression and OS of EGFR-mutant NSCLCs).

### 3.5 Subgroup analysis

Subgroup analysis was stratified by cutoff standards for PD-L1 positive, IHC assays and line of TKI-treatment (Table 2). Results of subgroup analysis indicated when 1% was used as cutoff value, PD-L1 positive group had a poor outcome including shorter PFS (HR [95% CI] = 1.97 [1.36–2.35], *P* < .001), OS (HR [95% CI] = 5.82 [1.09–30.96], *P* = .039) of EGFR-TKI treatment and OS (HR = 3.34, 95% CI: 1.31–8.56; *P* = .012) of overall EGFR-mutant cohort. Interestingly, PD-L1 positive group showed longer PFS (HR [95% CI] = 0.40, [0.25–0.64], *P* < .001) and OS (HR [95% CI] = 0.39 [0.17–0.87], *P* = .022) of EGFR-TKI therapy when assay ab58810 was applied for IHC assessment, while PFS (HR [95% CI] = 1.88 [1.38–2.57], *P* < .001) and OS (HR [95% CI] = 2.88 [1.58–5.26], *P* = .001) of EGFR- TKI therapy were shorter in PD-L1 positive group when assay 22C3 was applied. Furthermore, prolonged PFS was associated with negative PD-L1 expression when EGFR-TKIs were given as the first-line treatment (HR [95% CI] = 2.07 [1.16–3.68], *P* = .014). Subgroup analysis indicated none of the IHC assays, cutoff values or line of TKI-treatment were responsible for the heterogeneity.

**Table 2:** Results of subgroup analysis.

	ORR		PFS		OS1		OS2	
	Pooled OR [95% CI]	*I* ^2^	Pooled HR [95% CI]	*I* ^2^	Pooled HR [95% CI]	*I* ^2^	Pooled HR [95% CI]	*I* ^2^
Cutoff values								
1%	0.66 [0.15, 2.90]	84.8%	1.97 [1.36, 2.85]	22.8%	5.82 [1.09, 30.96]	56.9%	3.34 [1.31, 8.56]	50.3%
5%	0.56 [0.29, 1.07]	0%	0.56 [0.29, 1.07]	38.8%	0.56 [0.29, 1.06]	0%	0.88 [0.36, 2.15]	61.3%
IHC assays								
ab58810	**/**	**/**	0.40 [0.25, 0.64]	0%	0.39 [0.17, 0.87]	38.5%	**/**	**/**
E1L3N	**/**	**/**	**/**	**/**	**/**	**/**	1.22 [0.21, 7.22]	83.0%
22C3	**/**	**/**	1.88 [1.38, 2.57]	16.0%	2.88 [1.58, 5.26]	21.6%	1.86 [1.12, 3.07]	55.0%
SP142	**/**	**/**	2.37 [0.78, 7.17]	94.4%	**/**	**/**	1.29 [0.62, 2.68]	83.5%
Line of TKI treatment								
1st	0.51 [0.17, 1.56]	73.7%	2.07 [1.16, 3.68]	88.8%	1.24 [0.70,2.20]	45.4%	**/**	**/**
1st/≥2nd	0.57 [0.34, 0.96]	0.0%	1.08 [0.61, 1.91]	81.4%	1.34 [0.48, 3.79]	85.9%	**/**	**/**

CI = confidence interval, EGFR = epidermal growth factor receptor, HR = hazard ratio, ORR = objective response rate, OS = overall survival, OS1 = represents OS of EGFR-TKI treatment, OS2 = represents OS in EGFR-mutant cohort, PD-L1 = programmed death-ligand 1, PFS = progression-free survival, TKI = tyrosine kinase inhibitor.

## 4 Discussion

The interaction between oncogenic EGFR pathway and PD-1/PD-L1 axis is complicated and has been raising concerns in recent years. Although increasing evidences demonstrate NSCLCs with EGFR activation may predict a poorer response to immunotherapy of PD-1/PD-L1inhibitors, whether immune surveillance influence the efficacy of EGFR-TKIs remains unclear.^[30–33]^ Clarifying the relationship between PD-L1 expression and EGFR-TKI treatment may contribute to identify the NSCLC populations who would be most benefit from molecular-targeted therapy precisely. Before the presented study, a series of studies exploring the relationship between PD-L1 expression and EGFR-TKIs responsiveness have not reached a consistent conclusion. Thus, we conducted the presented meta-analysis to further investigate this issue.

Our study revealed a significant lower ORR in PD-L1 positive group, which indicated PD-L1 expression of NSCLC may involve in the mechanism of primary resistance to EGFR-TKIs. Sensitivity analysis suggested the heterogeneity may attribute to the study from Takashima et al (Figure S1, Supplemental Digital Content, http://links.lww.com/MD2/A338, sensitivity analysis of the relationship between PD-L1 expression and ORR of EGFR-TKI treatment), and heterogeneity was significant reduced when we deleted this study from the pooled result. We noted that the clinical characteristics of included cases among different PD-L1 status were not presented in their study. Lacking control and adjustment for confounding factors may probably lead to some potential bias in their results.

In terms of the PFS and OS of EGFR-TKI treatment, pooled HRs suggested predictive values of PD-L1 expression were limited. However, PD-L1 positive group had a poor outcome when 1% was used as a cutoff value for defining PD-L1 positive. Before our study, Bai et al also reported PD-L1 status did not significantly associate with PFS and OS of EGFR-TKI treatment in a meta-analysis.^[34]^ Interestingly, studies from Azuma et al and Akbay et al suggested inhibiting EGFR signaling with erlotinib could downregulate the expression of PD-L1 in EGFR-mutant NSCLC cell lines.^[35,36]^ Basing on the results of preclinical studies, EGFR-TKIs are supposed to inhibit tumor not only by the directly blocking EGFR signaling, but also by consequently restoring antitumor immune response such as PD-L1 downregulation. Apparently, the results of preclinical studies were inconsistent with the clinical data. We speculated that comparing to the effects of PD-L1 expression, effects of EGFR-TKIs on the EGFR signaling pathway may be more dominating in the NSCLC molecular-targeted therapy response. Although PFS and OS were significant shorter in the subgroup of 1% for PD-L1 positive, 5% was more commonly used as cutoff value in the included studies. Therefore, we look forward to further studies with 1% cutoff value to confirm our results. Although several studies assessing inter-assay concordance found high agreement between PD-L1 IHC assays, subgroup analysis basing on IHC assays indicated that diverse predictive values of PD-L1 expression in EGFR-TKIs efficacy among subgroups.^[37]^ Better outcome of EGFR-TKIs was shown in PD-L1 positive group when ab58810 was applied, while PFS and OS were significant shorter in PD-L1 positive group when 22C3 was applied. EGFR-TKI treatment given in first-line setting is now highly recommended for patients suffering advanced-stage EGFR-mutant NSCLC.^[38]^ However, a significant shorter PFS was found in PD-L1 positive group when EGFR-TKIs were given as the first-line treatment, which should deserve more attention within our clinical practice.

Moreover, we tried to initially evaluate the prognostic values of PD-L1 in EGFR-mutant NSCLCs. Several studies have reported the OS analysis in the overall population of NSCLC basing on PD-L1 status, and PD-L1 expression is generally considered as a prognostic factor related with poor survival.^[39,40]^ However, few studies focused on prognostic values of PD-L1 in EGFR-mutant population. Thus, the relationship between PD-L1 expression and outcome of EGFR-mutant NSCLCs is still unclear yet. From the pooled result of 13 studies (15 cohorts), we concluded that status of PD-L1 did not correlate with the prognosis of EGFR-mutant cohort, but the OS in PD-L1 positive group was also significant shorter when 1% cutoff value was applied. Accordingly, our study indicated 1% might be an important cutoff value to evaluate the effects of PD-L1 on outcome of EGFR-TKI treatment and prognosis of EGFR-mutant NSCLCs. In addition, when 22C3 assay was applied for IHC assessment, a significantly shorter OS was presented in EGFR-mutant NSCLCs with positive PD-L1 expression.

However, there were some limitations in our study. Firstly, prospective data were lacked and all the included studies were retrospective. Secondly, attributing to higher prevalence of EGFR mutation, most of included studies were conducted in East-Asian population, which makes the conclusions are heavily based on East-Asian population. In addition, existence of confounding factors resulted in heterogeneous quality of included data. For example, different generations of EGFR-TKI including erlotinib, gefitinib, and afatinib were used among included studies. Besides, gender, age, pathological type, smoking status, and diverse EGFR mutation status could also be potential confounding factors. The number of publications and the eligible data included in our meta-analysis was relatively small. Further investigation of this subject will be required to determine if PD-L1 expression level could act as a reliable predictor for EGFR-TKI therapy. Likewise, the prognostic values of PD-L1 expression in EGFR-mutant cohort are needed to be further evaluated.

## 5 Conclusion

In conclusion, positive PD-L1 expression indicated a higher incidence of primary resistance, but did not correlate with the PFS or OS of EGFR-TKI therapy. In addition, PD-L1 expression was unlikely a predictive biomarker for prognosis of EGFR-mutant NSCLCs.

## Author contributions

**Conceptualization:** Jingni Wu.

**Data curation:** Bo Lan, Yongfang Wang, Jingni Wu, Pingli Wang.

**Formal analysis:** Bo Lan, Jingni Wu.

**Investigation:** Bo Lan, Jingni Wu.

**Methodology:** Bo Lan, Yongfang Wang, Jingni Wu.

**Project administration:** Bo Lan.

**Resources:** Bo Lan, Yongfang Wang.

**Software:** Bo Lan, Yongfang Wang.

**Supervision:** Bo Lan, Kai Wang.

**Validation:** Bo Lan, Jingni Wu, Kai Wang, Pingli Wang.

**Visualization:** Bo Lan, Yongfang Wang, Jingni Wu, Kai Wang, Pingli Wang.

**Writing – original draft:** Bo Lan, Jingni Wu, Kai Wang, Pingli Wang.

**Writing – review & editing:** Bo Lan, Jingni Wu, Kai Wang, Pingli Wang.
